# Seizure Suppression by High Temperature via cAMP Modulation in *Drosophila*

**DOI:** 10.1534/g3.116.034629

**Published:** 2016-08-23

**Authors:** Arunesh Saras, Mark A. Tanouye

**Affiliations:** * Department of Environmental Science, Policy and Management, University of California, Berkeley, California 94720; † Department of Molecular and Cell Biology, University of California Berkeley, California 94720

**Keywords:** sodium channel, epilepsy, seizure suppression, *rutabaga*, cAMP, FlyBook

## Abstract

Bang-sensitive (BS) *Drosophila* mutants display characteristic seizure-like activity (SLA) and paralysis after mechanical shock . After high-frequency electrical stimulation (HFS) of the brain, they generate robust seizures at very low threshold voltage. Here we report an important phenomenon, which effectively suppresses SLA in BS mutants. High temperature causes seizure suppression in all BS mutants (*para^bss1^*, *eas*, *sda*) examined in this study. This effect is fully reversible and flies show complete recovery from BS paralysis once the temperature effect is nullified. High temperature induces an increase in seizure threshold after a brief pulse of heat shock (HS). By genetic screening, we identified the involvement of cAMP in the suppression of seizures by high temperature. We propose that HS induces adenylyl cyclase which in turn increases cAMP concentration which eventually suppresses seizures in mutant flies. In summary, we describe an unusual phenomenon, where high temperature can suppress SLA in flies by modulating cAMP concentration.

*Drosophila* bang-sensitive (BS) mutants with epilepsy-like phenotypes are used as an animal model to study seizure disorder. BS mutants are known phenotypically for their characteristic seizure-like activity (SLA) and paralysis behavior. In adult stage BS mutants, SLA can be induced by mechanical shock or evoked by high-frequency electrical stimulation (HFS). The behavioral phenotype is complex with several distinguishable phases: seizure, initial paralysis, tonic–clonic-like activity and recovery seizure (Benzer 1971; Ganetzky and Wu 1982; Pavlidis *et al.* 1994; Pavlidis and Tanouye 1995; Lee and Wu 2002; Zhang *et al.* 2002; Parker *et al.* 2011a,b; Burg and Wu 2012).

Numerous BS genotypes have been reported including *easily shocked (eas)*, *slamdance (sda)*, and *paralytic^bss1^* (*para^bss1^)* (Pavlidis *et al.* 1994; Zhang *et al.* 2002; Parker *et al.* 2011a,b). The *para^bss1^* mutant is the strongest seizure-prone mutant and hardest to suppress by suppressor mutation and drug, and has been presented as a model for human intractable epilepsy. It is a gain-of-function mutation of the *Drosophila* voltage-gated Na^+^ channel that resembles especially the human SCN2A mutation p.Arg1882Gly responsible for neonatal epilepsy with late-onset episodic ataxia (Parker *et al.* 2011a,b; Schwarz *et al.* 2016).

BS mutants also represent a unique class of temperature-sensitive paralysis mutants. Recently it has been shown that BS mutants also display temperature-induced seizures upon exposure to either low or high temperature; however, different brain anatomical foci are required for seizure induction by either mechanical or temperature stressors (Burg and Wu 2012). In particular, *eas* and *para^bss1^* undergo seizures by exposure to low temperature (8°) whereas *bang-sensitive* (*bas*) and *technical knock-out* (*tko*) exhibited temperature-induced-seizures at high temperature (39°). The allele *para^GEFS+^* gives rise to temperature-dependent SLA (Sun *et al.* 2012). Several loss-of-function *para* alleles, including *para^ts1^* and *para^ST76^*, are conditional temperature-sensitive paralytic mutations causing immediate, but reversible paralysis of adult flies when they are shifted from permissive to restrictive temperature (Suzuki and Grigliatti 1971; Siddiqui and Benzer 1976; Ganetzky and Wu 1982; Lee and Wu 2002; Burg and Wu 2012). In another temperature-sensitive mutant, *mle^napts^* (maleless no action potential, temperature-sensitive) (Kernan *et al.* 1991), axonal conduction fails at high temperature. Synaptic transmission is impaired at restrictive temperatures, which leads to rapid paralyses in larvae and adults (Wu *et al.* 1978).

In *Drosophila*, various other channel types and second messengers are also involved in temperature-sensitive behavior, which include not only transient receptor potential (TRP) ion channels but also cAMP. For example, *painless*, one of the TRP channel superfamily members, is required for sensing nociceptive stimuli at temperatures over 38° (Tracey *et al.* 2003). Pyrexia, another TRP channel, protects flies from high temperature stress over 40° (Lee *et al.* 2005). In contrast, dTRPA1, a heat-activated TRP family ion channel, participates in the temperature selection by opening at warm temperatures (24–29°) (Rosenzweig *et al.* 2005). Moreover, in mushroom bodies (MB), cAMP signaling modulates temperature preference behavior (TPB) in *Drosophila* (Hong *et al.* 2008).

The adenylyl cyclases (ACs) are enzymes with key regulatory roles in all cells. ACs catalyze the conversion of adenosine triphosphate (ATP) to 3′,5′-cyclic AMP (cAMP). In *Drosophila* the best studied AC is *rutabaga* (*rut*) which encodes a Ca^2+^/calmodulin-responsive adenylyl cyclase (Levin *et al.* 1992). Flies carrying the *rut^1^* allele have low levels of adenylyl cyclase activity, especially Ca^++^/CaM-stimulated activity (Livingstone *et al.* 1984; Dudai *et al.* 1985). cAMP is a second messenger, and used for intracellular signal transduction in numerous physiological and developmental processes, including associative learning (Quinn *et al.* 1974; Livingstone *et al.* 1984).

In the present study, using behavioral assays and electrophysiology, we describe an unusual phenomenon where high temperature causes suppression of seizures. We find that at 38°, SLA is reduced in *Drosophila* BS mutants. This suppressive effect of temperature requires only a brief pulse of HS, with the effect fully reversible when flies are returned back to room temperature. We show that high-temperature seizure suppression depends on cAMP: suppression does not occur under conditions of low [cAMP] due to adenylyl cyclase loss-of-function.

## Materials and Methods

### Fly stocks

*Drosophila* strains were maintained on standard cornmeal–molasses–agar medium at room temperature (24°). The *paralytic* (*para*) gene is located at map position 1–53.5 and encodes a voltage-gated Na^+^ channel (Loughney *et al.* 1989; Ramaswami and Tanouye 1989). The allele used here is the bang-sensitive (BS) paralytic mutation, *para^bss1^*, previously named *bss^1^*. It is the most seizure-sensitive of fly mutants, the most difficult to suppress by mutation and by drug, and is a model for human intractable epilepsy (Ganetzky and Wu 1982; Parker *et al.* 2011a,b). The *para^bss1^* allele is a gain-of-function mutation caused by a substitution (L1699F) of a highly conserved residue in the third membrane-spanning segment (S3b) of homology domain IV (Parker *et al.* 2011a,b). The *easily shocked* (*eas*) gene is located at 14B on the cytological map and encodes an ethanolamine kinase (Pavlidis *et al.* 1994). The BS allele used in this study is *eas^PC80^*, which is caused by a 2-bp deletion that introduces a frame shift; the resulting truncated protein lacks a kinase domain and abolishes all enzymatic activity (Pavlidis *et al.* 1994). The *slamdance* (*sda*) gene is located at 97D and encodes an aminopeptidase N. The allele used in this study is *sda^iso7.8^* caused by a 2-bp insertion in the 5′ untranslated region (Zhang *et al.* 2002). The *rutabaga (rut)* is located at 12F5-7 and encodes an adenylyl cyclase (Livingstone *et al.* 1984; Levin *et al.* 1992). The allele used in this study, *rut^1^*, is a loss-of-function mutation caused by an amino acid substitution (G1026R; Levin *et al.* 1992). The *rut^1^* mutant flies have low levels of adenylyl cyclase activity, especially Ca^++^/CaM-stimulated activity (Livingstone *et al.* 1984). The *rut^1^* and *UAS-rutRNAi* lines were obtained from the Bloomington *Drosophila* Center. The insert for *UAS-rutRNAi* is located on the 3rd chromosome.

### BS behavior and HS

Behavioral testing for BS paralysis was performed on flies 3 d after eclosion, as described previously (Kuebler and Tanouye 2000). Flies were anesthetized with CO_2_ before collection and tested the following day. For testing, 10 flies were placed in a clean food vial and stimulated mechanically with a VWR vortex mixer at maximum speed for 10 sec. The *para^bss1^*, *eas*, and *sda* mutants ordinarily show 100% penetrance of BS \paralytic behavior. Recovery from BS paralysis was determined by counting the number of flies standing at different intervals following stimulation. Recovery time was the time where 50% of flies had recovered. For genotypes that display partial penetrance of BS paralysis, only those flies that displayed paralysis were used for recovery time analysis. For BS behavioral analysis, pools of flies are combined for each genotype from among the separate trials (in total, *n* ≈ 100 for each genotype). For analyses using HS, 10 flies were placed in a clean food vial and tested the following day. The vial was submerged in a water bath (38° for 30 sec), and then tested for BS behavioral paralysis. The time between HS and behavioral testing was typically 30 sec.

### Electrophysiology

*In vivo* recording of SLA and seizure threshold determination in adult flies was performed as described previously (Kuebler and Tanouye 2000; Lee and Wu 2002). Flies 2–3 d posteclosion were mounted in wax on a glass slide, leaving the dorsal head, thorax, and abdomen exposed. Stimulating, recording, and ground metal electrodes were made of uninsulated tungsten. Seizure-like activity was evoked by high-frequency electrical brain stimulation (0.5-msec pulses at 200 Hz for 300 msec) and monitored by dorsal longitudinal muscle (DLM) recording. During the course of each experiment, the giant fiber (GF) circuit was monitored continuously as a proxy for holobrain function. For seizure-threshold determination in HS flies, one fly/food vial was placed and tested the following day. Prior to recording, they were HS for 30 sec at 38° and mounted in wax on a slide and used for electrophysiological recordings. The time between delivery of the HS and electrophysiological recording was typically 2–3 min. For each genotype tested *n* ≥ 5.

### Data analysis

All error bars shown represent the standard error of the mean. Chi-square tests were used to compare the penetrance of seizures. Student’s *t*-test and ANOVA were used to compare recovery times and seizure thresholds across genotypes, as appropriate. For ANOVA analysis, where the null hypothesis was rejected by the overall *F* ratio, multiple comparisons of data sets were performed by Fisher’s least significant difference with *t*-test significance set at *P* < 0.05.

### Data availability

The authors state that all data necessary for confirming the conclusions presented in the article are represented fully within the article.

## Results

### High temperature suppresses seizures in BS mutants

**Figure 1:**
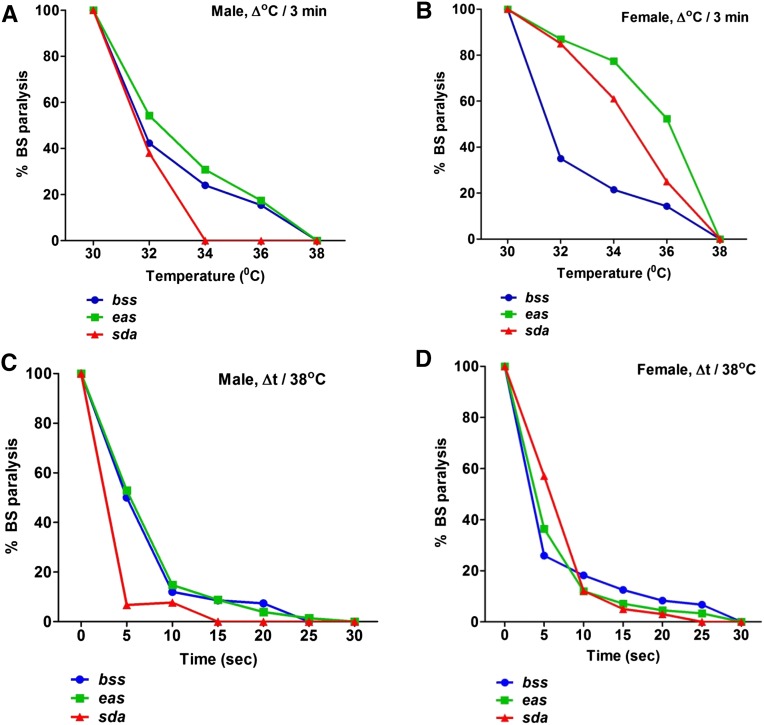
High temperature suppresses behavioral paralysis in BS mutants. Effects of different temperatures on behavioral paralysis in BS males (A) and females (B). HS was applied for 3 min at different temperatures from 30 to 38°. BS paralysis was correspondingly decreased from lower to higher temperature indicating increasing suppression. In all genotypes, complete suppression of BS paralysis is seen at 38°. Effects of different HS durations on behavioral paralysis in BS males (C) and females (D). HS pulses of 38° and varying durations ranging from 5 sec to 3 min were delivered to BS flies. BS paralysis for all flies was completely suppressed by 30 sec HS pulses. For each data point *n* ≥ 50 flies.

To investigate the effect of temperature on seizure-sensitivity in BS mutants, we used a brief HS protocol. The BS mutants were HS at 32° for 3 min in the water bath. We found that BS paralysis behavior in *para^bss1^* mutants was decreased in both males and females up to 40% compared to control non-HS *para^bss1^* mutants, which showed 100% BS paralysis. To test if HS decreases BS paralysis in other BS mutants, we checked *eas* and *sda* mutants under the same HS protocol. We observed a significant decrease in BS paralysis in both *eas* and *sda* mutants. In *eas*, BS paralysis was decreased to 60–80% (males and females) whereas in *sda*, it was reduced to 40–80% (in males and females) compared to 100% BS at room temperature (Figure 1, A and B). Thus, regardless of genotype, HS suppresses BS paralysis in all BS mutants studied here.

### Dependence of seizure suppression by HS on temperature and time

We sought to determine the optimum conditions for HS at which maximum seizure suppression can be achieved in all BS mutants examined in this study. We hypothesized that both the range of temperature and time duration for HS may affect the seizure susceptibility. To determine the optimum temperature for HS to maximally suppress BS paralysis, we used the same HS protocol but varied the range of temperature for HS. HS was given that varied from 30 to 38° temperature range for 3 min. We found a strong correlation between temperature and reduction in BS paralysis. BS paralysis was correspondingly decreased from lower to higher temperature. All the BS mutants at room temperature exhibited 100% BS paralysis behavior, but from 30 to 38° BS paralysis behavior was decreased from 100 to 0% (Figure 1, A and B). At 38° BS paralysis was decreased to ∼0% compared to controls without HS; therefore, we decided to perform subsequent experiments using HS at 38°.

Next we varied our HS protocol to determine the optimum time duration dependence of HS. HS was given at 38° for different times, ranging from 10 sec to 3 min. We found that the reduction in BS paralysis was strongly dependent on the time duration of HS. From 10 sec to 3 min of HS at 38°, we observed a decrease in BS paralysis from 100 to 0% (Figure 1, C and D). Maximum reduction in BS paralysis was observed by 30 sec and prolonging the HS duration up to 3 min did not change BS paralysis significantly (Figure 1, C and D). From these results, we concluded that HS at 38° with a time duration of 30 sec was sufficient to achieve maximum suppression in BS paralysis in BS mutants. Hence, in *para^bss1^*, *eas*, and *sda* mutants flies, BS paralysis was 0% (complete suppression) after HS. There is no gender bias in BS paralysis suppression in *para^bss1^*, *eas*, and *sda* BS mutants as both males and females show a reduction in BS paralysis (Figure 1, C and D).

### Duration of HS-induced BS paralysis suppression (HIBPS) in BS mutants

**Figure 2:**
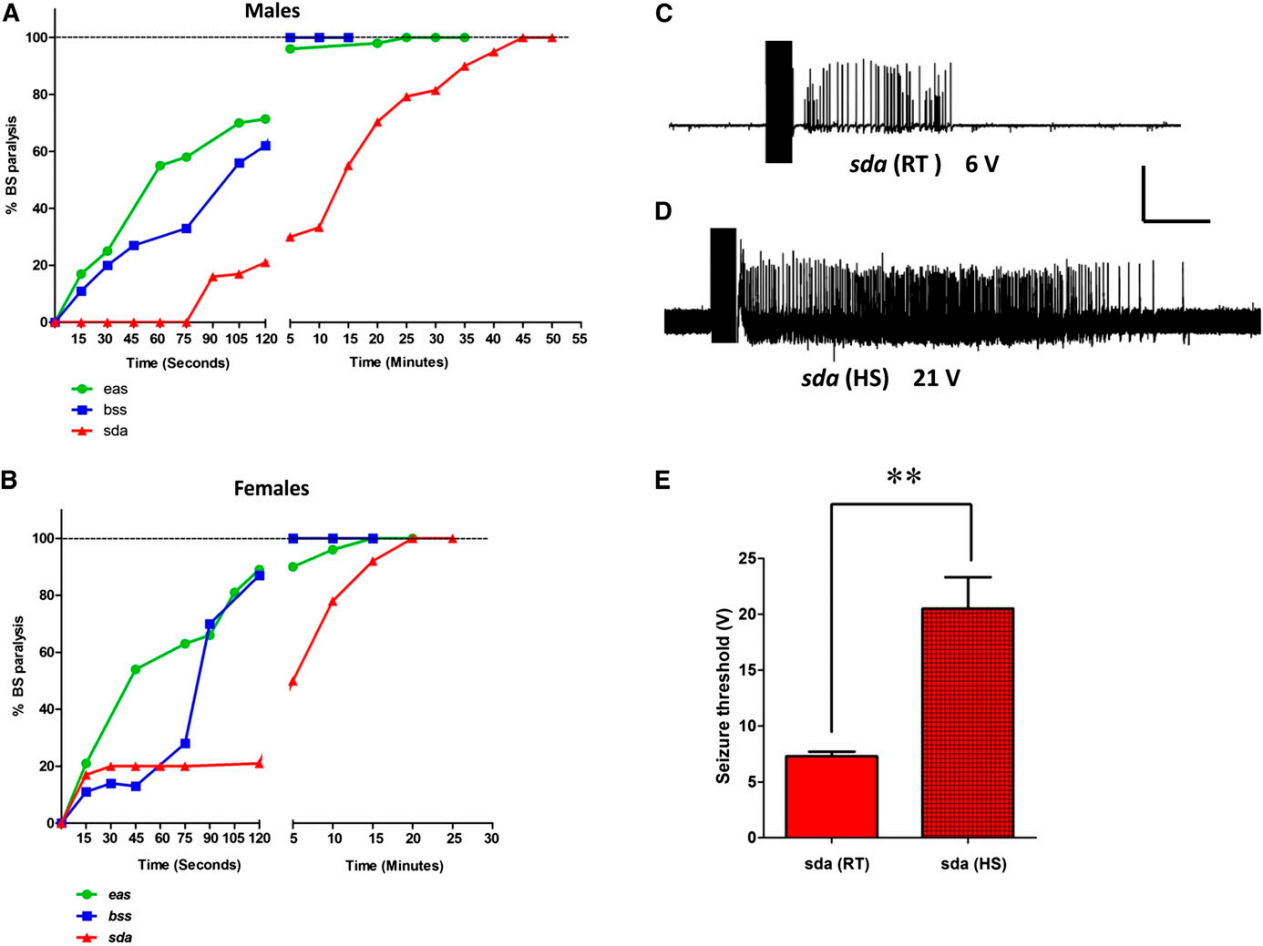
Duration of HS-induced BS paralysis suppression (HIBPS) in BS mutants, and electrophysiology of seizure thresholds. (A) Seizure suppression decreases with time after delivery of the HS. HIBPS decrease for *eas* and *para^bss^* males is substantial within 2 min of HS. In contrast, single mutant *sda* males take longer than 30 min to lose HIBPS. (B) HIBPS decreases more rapidly in BS females than males, especially *para^bss1^* and *eas* females. For each data point *n* ≥ 30 flies. (C) Electrophysiological recording from a *sda* DLM fiber evoked by a 6 V HFS stimulation (0.5 msec stimuli at 200 Hz for 300 msec) at room temperature. The stimulation evokes seizure-like electrical activity (SLA), indicating that the voltage is at or above seizure threshold. (D) Electrical recording from a *sda* DLM fiber following HS (38° for 30 sec). A HFS stimulation voltage of 21 V HFS is required to evoke SLA. There has been an increase in seizure threshold caused by the HS. Horizontal calibration is 1 sec for (C and D). Vertical calibration is 20 mV. (E) Seizure threshold in *sda* at room temperature is 7.3 ± 0.41 V HFS, *n* = 5. Following HS, *sda* seizure threshold is substantially greater: 20.5 ± 2.81 V HFS, *n* = 7.

To investigate how long HIBPS persists in BS mutants, we analyzed BS paralysis behavior in BS mutants at different time intervals following HS, ranging from 15 sec to 1 hr. We found that HIBPS decreased with longer time duration and displayed genotype bias. In *para^bss1^* and *eas* BS male and female mutants, nearly 80% of flies showed a decrease in HIBPS within 2 min, whereas in *sda* this decrease in HIBPS was slower and required at least 20 min. Also, *sda* mutants displayed gender-biased BS paralysis behavior, with female mutants showing a faster decrease in HIBPS (100% BS after 20 min) compared to male mutants (100% BS after 45 min) (Figure 2, A and B). Therefore, these findings indicate that HIBPS is dependent on time interval and BS mutants show complete reversion in HIBPS within an hour after the HS.

### HS alters seizure threshold in BS mutants

Seizure threshold (the minimum voltage of an HFS required to induce a seizure) is a quantitative measure of a fly’s seizure susceptibility. In particular, seizure-sensitive BS mutants have a low seizure threshold (Kuebler and Tanouye 2000; Lee and Wu 2002; Zhang *et al.* 2002; Parker *et al.* 2011a,b) whereas a seizure suppressor usually increases the seizure threshold in BS mutants (Kuebler *et al.* 2001; Lee and Wu 2006; Song and Tanouye 2006, 2007; Song *et al.* 2007; Howlett and Tanouye 2013). To check if HS alters the seizure threshold in BS mutants, we performed electrophysiological recordings to determine threshold in BS mutants after HS treatment. We stimulated concurrently the GF circuit using single pulse stimulation and recorded evoked DLM potentials. The GF circuit acts as a proxy for the state of the entire nervous system during and after SLA. Since in *para^bss1^* and *eas* mutants HIBPS reverts back very quickly (within 5 min), it was not feasible to determine the effect of HS on seizure threshold in these mutants. Therefore, we used *sda* single mutants to study the effect of HS on seizure threshold. HS significantly changed the seizure threshold for seizures in *sda*. At room temperature, SLA can be induced in *sda* at low seizure threshold (7.3 ± 0.41 V HFS, mean ± SEM, *n* = 5; *P* < 0.0001, ANOVA test; Figure 2C and E). However, after HS, the seizure threshold became nearly threefold higher (20.5 ± 2.81 V HFS, mean ± SEM, *n* = 8; *P* < 0.0001, ANOVA test; Figure 2D and E). Thus, suppression of BS paralysis appears to be due to an increase in seizure threshold after HS in BS mutants.

### Reverse genetic RNA interference (RNAi) screening to identify the underlying mechanism in the suppression of BS by HS

To elucidate the molecular mechanism of seizure suppression by HS, we carried out reverse genetic RNAi screening. We hypothesized that any identified mutant that abolishes the HIBPS, *i.e.*, even after HS flies show BS paralysis, should be involved in the seizure suppression mechanism. Using the GAL4/UAS binary system, in particular *elav^c155^-GAL4 para^bss1^* and UAS-RNA*i* lines, 14 genes were screened (*pyrexia* (*pyx*), *nanchung* (*nan*), *transient receptor potential cation channel A1 ortholog* (*dTrpA1*), *synatobrevin* (*Syb*), *synptosomal-associated protein 24kDa* (*Snap24*), *synptosomal-associated protein 25kDa* (*Snap25*), *Rab3 interacting molecule* (*Rim*), *Rim binding protein* (*Rbp*), *syntaxin 1A* (*Syx1A*), *painless* (*pain*), *histamine-gated chloride channels subunit 1*(*hisCl1*), *ora transientless* (*ort*), *cyclic nucleotide-gated ion channel-like* (*cng*), and *I_h_ channel* (*I_h_*). These genes were selected based on the assumption that genes involved in thermoregulation or synaptic transmission might be involved in HIBPS. However neither approach produced any reversion in BS paralysis by HS.

**Figure 3:**
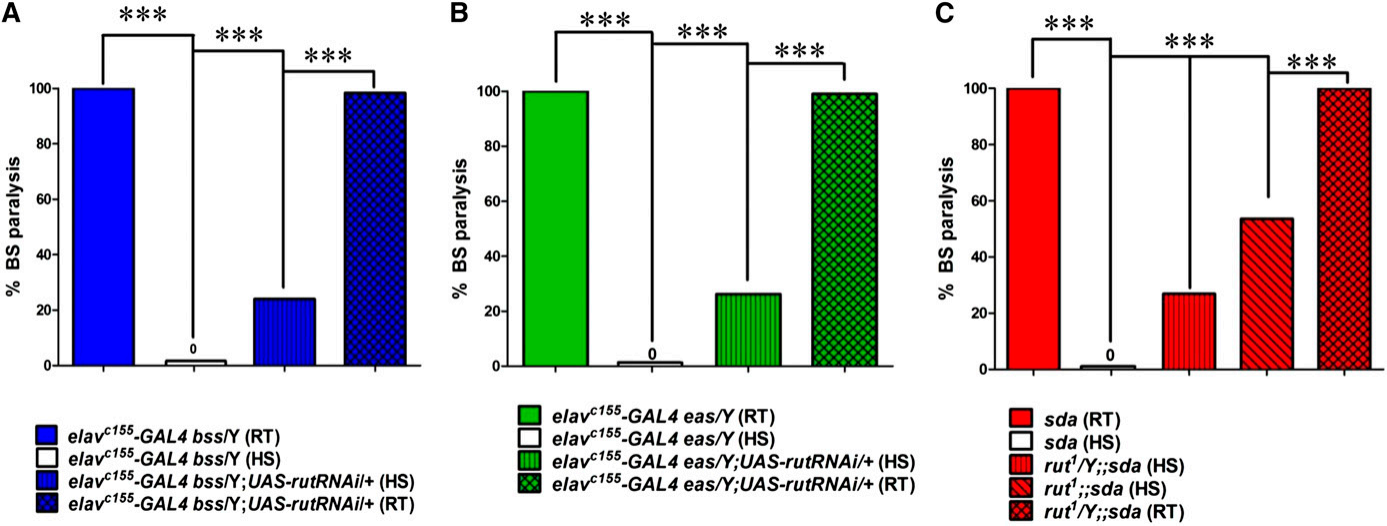
HIBPS in BS mutants requires *rutabaga* function. (A) Pan-neuronal expression of *UAS-rut RNAi* reduces the HS-induced suppression in BS paralysis behavior. In double mutants [blue striped bar, genotype *elav^c155^-GAL4 para^bss1^/*Y*;;UAS-rutRNAi/*+ (HS)], expression of *rutRNAi* driven by *elav-GAL4* produces 24% BS paralysis following HS (*n* = 120) compared to 0% BS paralysis in control *elav^c155^-GAL4 para^bss1^/Y* hemizygous males (*n* = 80, white bar) after HS. Flies with same genotype [blue crosshatched bar, *elav^c155^-GAL4 para^bss1^/*Y*;;UAS-rutRNAi/*+ (RT)] at RT showed 100% BS paralysis (*n* = 120). (B) Pan-neuronal expression of *UAS-rutRNAi* also reverts the HS-induced suppression in *eas* paralysis behavior. Double mutants [green striped bar, genotype *elav^c155^-GAL4 eas*/Y;;*UAS-rut RNAi/*+ (HS)] displayed 26.2% BS paralysis behavior (*n* = 42) following HS compared to 0% BS paralysis in single hemizygous mutants *elav^c155^-GAL4 eas*/Y (*n* = 50, white bar). Flies with same genotype [green crosshatched bar, genotype *elav^c155^-GAL4 eas*/Y;;*UAS-rut RNAi/*+(RT)] at RT showed 100% BS paralysis (*n* = 45). (C) HIBPS is reduced in *sda* by the *rut^1^* mutant. Hemizygous male double mutants (genotype, *rut^1^*/Y;;*sda*) show 27% BS paralysis following HS (*n* =502, red vertically striped bar, *P* ≤ 0.0001, Fisher’s exact test). Double mutant *rut^1^;;sda* female flies show 54% BS paralysis following HS (*n* = 398, red diagonally striped bar), whereas at RT flies with the same genotype (*rut^1^;;sda*) were 90% BS (*n* = 209, red crosshatched bar).

Recently, it has been shown that in the *Drosophila* MB, cAMP signaling modulates TPB (Hong *et al.* 2008). Flies with low levels of cAMP prefer a lower temperature and flies with high levels of cAMP prefer a high temperature. We tested whether the cAMP signaling pathway may be involved in BS paralysis suppression by HS. When *elav^c155^-GAL4 para^bss1^* female flies were crossed with *UAS-rut RNAi* males, the double mutant males with genotype *elav^c155^-GAL4 para^bss1^*/Y;;*UAS-rut RNAi/+* showed a significant increase in BS paralysis after HS. These double mutants showed 24% (*n* = 120; *P* ≤ 0.0001, Fisher’s exact test; Figure 3A) BS paralysis after HS compared to control *elav^c155^ para^bss1^*/Y hemizygous males with ∼0% BS paralysis after HS. To further confirm the effect of *UAS-rutRNAi* in different BS genotype, we crossed *elav^c155^–GAL4 eas* female flies with *UAS-rutRNAi* males. The double mutant males with genotype *elav^c155^-GAL4 eas*/Y;;*UAS-rut RNAi/+* also showed a significant increase in BS paralysis after HS. These double mutants showed 26.2% (*n* = 42; *P* ≤ 0.0001, Fisher’s exact test; Figure 3B) BS paralysis after HS compared to control *elav^c155^ eas*/Y hemizygous males with ∼0% BS. From this genetic screening, we isolated *rut* as a possible candidate gene and concluded that *rut*, and thus cAMP, might be involved in HIBPS in BS mutants.

### Reversion of HIBPS of BS paralysis by rut^1^ mutant

To further explore and to validate *rut* as the gene involved in suppression of BS paralysis by HS, we used the *rut^1^* mutant. We generated double mutant *rut^1^;;sda* flies by crossing *rut^1^* mutant in the *sda* background. When *rut^1^;;sda* double mutants are HS, we observed a significant increase in BS paralysis. The BS paralysis after HS was ∼40% (including both males and females) (*n* = 900; *P* ≤ 0.0001, Fisher’s exact test; Figure 3C) compared to control mutant *sda* single mutants which showed 0% BS paralysis after HS. However, we observed some gender-biased change in BS paralysis. *rut^1^;;sda* double mutant females showed a higher increase in BS paralysis after HS than males. BS paralysis in *rut^1^;;sda* double mutant female flies was ∼54% (*n* = 398) after HS, whereas in double mutant *rut^1^/y;;sda* male flies BS paralysis was only 27% (*n* = 502, Figure 3C). Taken together, these results indicate that *rut^1^* is involved in suppression of BS paralysis by HS.

**Figure 4:**
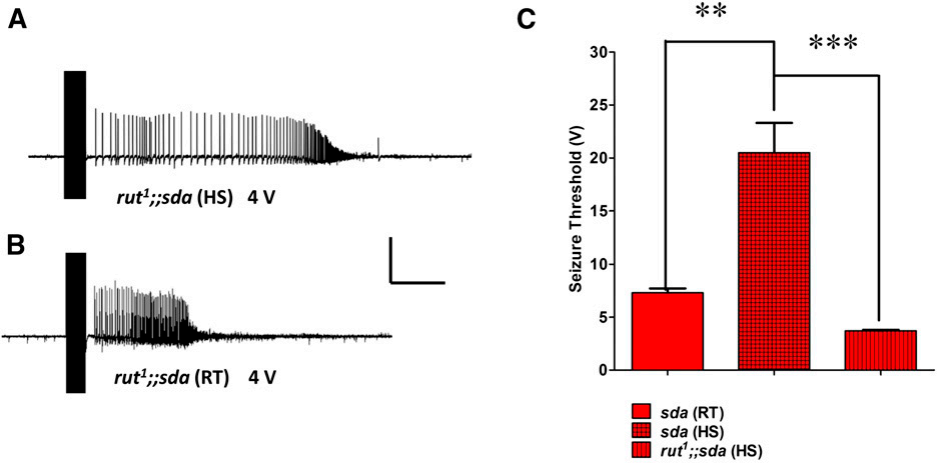
Electrophysiology of seizure thresholds in *sda* and *rut^1^* backgrounds after HS. (A) Electrophysiological recording from a *rut^1^;;sda* fly DLM fiber evoked by a 4 V HFS stimulation (0.5-msec stimuli at 200 Hz for 300 msec) following HS. The stimulation evokes seizure-like electrical activity (SLA), indicating that the voltage is at or above seizure threshold. (B) At room temperature, a *rut^1^;;sda* double mutant fly exhibits the same seizure threshold (4 V HFS). Horizontal calibration is 1 sec for (A–C), Vertical calibration is 20 mV. (C) Histogram compares seizure thresholds in *sda* and *rut^1^;;sda* genotypes. Seizure threshold is low in *sda* (RT) control flies (red bar, 7.3 ± 0.41 V HFS, *n* = 5). Following HS, *sda* seizure threshold is substantially increased (red checkered bar, 20.5 ± 2.81 V HFS, *n* = 7). In the double mutant *rut^1^;;sda* seizure threshold following HS is low (red striped bar, 3.7 ± 0.1 V HFS, *n* = 5) even after the HS.

Next, we sought to determine the change in seizure threshold due to *rut^1^* in *rut^1^;;sda* double mutants. Since *rut^1^* decreased the suppression of BS paralysis by HS, we anticipated that *rut^1^;;sda* double mutants should show a reduction in seizure threshold after HS compared to HS *sda* single mutants. We used electrophysiological recordings to determine seizure threshold for *rut^1^;;sda* double mutants and *sda* single mutant flies after HS. The seizure threshold for *rut^1^;;sda* double mutant flies was reduced sixfold (3.7 ± 0.1 V HFS, mean ± SEM, *n* = 5; similar to *sda* single mutants at room temperature) compared to *sda* single mutant HS flies (20.5 ± 2.81 V HFS, mean ± SEM, *n* = 8; *P* < 0.0001, unpaired Student’s *t*-test; Figure 4).This reduction in seizure threshold is correlated with the reversal of BS paralysis to the normal level. Thus, *rut* and hence cAMP may be involved in seizure suppression by HS in BS mutants by reducing the seizure threshold.

## Discussion

Human seizure phenotypes arise from interactions between genetic and environmental factors (Ottman *et al.* 1996; Szyf 2015). Every individual has the potential to have a seizure; however, some are more likely to have seizures than others. These individuals have a low level of resistance to seizures: a low seizure threshold. Seizure threshold is thought to be mainly due to genetic factors coming from familial inheritance (Kjeldsen *et al.* 2001; Peljto *et al.* 2014), but environmental factors can also modulate seizure threshold and make seizures more likely, for example severe head injury or infection (Ottman *et al.* 1996; Szyf 2015). Numerous environmental stresses are found to enhance seizure-sensitivity or trigger seizure events. For example, seizures can result from exposure to drugs and poisons, such as antidepressants, lead poisoning, nicotine, and carbon monoxide. Other environmental stresses triggering seizures include stress, lack of sleep, alcohol consumption, light flashes, and hormonal imbalance (Herman 2002; Wood *et al.* 2004; Jett 2012; Alexandre *et al.* 2015). Although genetic and environmental factors are both known contributors to seizure phenotypes, little is known about the mechanisms responsible for how they might interact.

*Drosophila* is attractive for evaluating genetic and environmental contributions to seizure susceptibility. The biggest advantage comes from a good collection of neurological mutations with known and quantifiable effects on seizure susceptibility that includes seizure-sensitive, seizure-resistant, seizure-suppressor, and seizure-enhancer mutations. Thus, genetic background can be well controlled with single mutants and double mutant combinations with all individuals of a given genotype having similar seizure thresholds. The present study examined the effect of temperature, as an environmental stressor, on seizure susceptibility in *Drosophila*. We find here and in two recent studies (Kroll *et al.* 2015; Saras and Tanouye 2016) that interactions between temperature and genetic background are complex in their effects on seizure susceptibility, and differ depending on the different mutations contributing to the genotype.

We show here that high temperature can indeed alter BS mutant seizure susceptibility. However, our initial assumption was found to be incorrect; this environmental stressor does not exacerbate seizure sensitivity. Instead of promoting or enhancing BS phenotypes, high temperature acts as a suppressor. BS paralytic behaviors are significantly reduced following HS in all three BS mutants examined in this study (*para^bss1^*, *eas*, and *sda*). HIBPS of *para^bss1^* is especially notable because its phenotypes have proven difficult to suppress by suppressor mutations and drugs (Parker *et al.* 2011a,b). Only a brief pulse of HS is required for HIBPS. When the flies are allowed to recover at room temperature, HIBPS itself quickly reverts and flies regain bang-sensitivity indicating that seizure suppression is transient.

Suppression of behavioral BS paralysis by HS appears to result from an increase in seizure threshold. During the period corresponding to HIBPS for *sda*, there is a transient increase in seizure threshold of about threefold as observed in electrophysiological recordings. Because of this seizure threshold increase, *sda* seizure-sensitivity, ordinarily low at room temperature, is brought close to the wild-type range. This is sufficient to account for the loss of the BS paralytic behavior for *sda*. Thus, we suggest that HS from the environment is capable of interacting with the *sda* nervous system; this interaction causes an increase in seizure threshold and a suppression of BS mutant phenotypes.

A challenge is to determine mechanisms for how environmental factors, such as high temperature, interact with the nervous system to alter seizure threshold. For this, genetic screening in *Drosophila* provides a promising approach. We can utilize mutations to gain molecular access to the challenge and use continued genetic analysis to elucidate the mechanism. We undertook genetic screening using RNAi in our initial attempt to identify an interaction mechanism for HIBPS. RNAi for candidate genes involved in thermoregulation or synaptic transmission were tested and found not to be effective in altering HIBPS.

In contrast, RNAi for the adenylyl cyclase gene, *rut*, resulted in significant levels of bang-sensitivity following HS. A role for *rut* in bang-sensitivity for this system is evident since *rut RNAi* was similarly effective for two BS mutants *para^bss1^* and *eas*; and the *rut^1^* mutation was effective for *sda*. Furthermore, electrophysiology shows that the change in HIBPS is correlated with a decrease in seizure threshold. This approximately sixfold decrease in seizure threshold is sufficient in magnitude to completely account for the change in HIBPS affected by *rut* loss-of-function. Thus, taken together our findings support a scenario whereby high temperature causes a suppression of BS phenotypes: behavioral paralysis and SLA, by increasing the seizure threshold of seizure-sensitive mutants. This suppression is dependent on intact adenylyl cyclase functionality and by inference on the up-regulation of cAMP signaling. What remains unclear is what the entire signaling pathway from HS to seizure threshold increase looks like, and exactly where in this pathway cAMP is required. There are attractive possibilities. For example, mammalian brain Na^+^ channels are regulated by cAMP-dependent protein kinase PKA (Scheuer 2011). In rat striatal neurons, Na^+^ channel phosphorylation can reduce inward currents thereby resulting in lowered neuronal excitability (Surmeier *et al.* 1992; Schiffmann *et al.* 1996). Seizure activity has been found to result in alterations in Na^+^ channel phosphorylation (Baek *et al.* 2014). These and other interesting possible mechanisms for interactions between environmental factors and SLA may be resolved in future investigations. Nevertheless, the discovery of cAMP in the pathway is a significant advance, and in subsequent analyses, we should be able to make progress in defining other key players.
